# Differences in Chronic Low-Grade Inflammation and Metabolic Disturbances between *VDR* Genotypes in an Ethnically Homogenous Postmenopausal Female Population from Poland

**DOI:** 10.3390/nu15122737

**Published:** 2023-06-13

**Authors:** Anna Bohdanowicz-Pawlak, Felicja Lwow

**Affiliations:** 1 Department of Endocrinology, Diabetology and Isotope Therapy, Wroclaw Medical University, Pasteur 4, 50-367 Wroclaw, Poland; 2 Department of Massage and Physical Therapy, Faculty of Physiotherapy, Wrocław University of Health and Sport Sciences, Paderewskiego 35, 51-612 Wroclaw, Poland; felicitas1@wp.pl

**Keywords:** *VDR* gene polymorphism, cytokines, metabolic disturbances, menopause

## Abstract

(1) Vitamin D deficiency and changes in the endocrine system may stimulate systemic inflammation. VDR expression and the vitamin D concentration decrease with age, which is important in postmenopausal women for whom estrogen deficiency causes rapid bone loss. This group is, moreover, particularly at risk of developing atherosclerosis and its adverse consequences, such as chronic inflammation. The aim of this study was to assess the differentiation by the *VDR* genotype of the risk factors for so-called chronic low-grade inflammation and metabolic disorders. (2) We studied the differences between the anthropometric, metabolic, and inflammation parameters of *VDR* genotypes for *Apa-I*, *Bsm-I*, *Fok-I*, and *Taq-I* in a sample of 321 women aged 50–60 from an ethnically homogeneous urban population in Poland. (3) The TT *Taq-I* genotype presented a significantly higher rate of insulin resistance (HOMA) and lower serum levels of adiponectin than the other two genotypes. The AA genotype of the *Bsm-I* polymorphism was associated with a more atherogenic serum profile and significantly higher LDL and LDL/HDL values and Castelli Index. (4) Chronic low-grade inflammation was associated with the TT *Taq-I* genotype and presented a higher rate of insulin resistance. The AA genotype of the *Bsm-I* polymorphism presented a more atherogenic serum lipid profile and, therefore, a higher risk of developing cardiovascular disease.

## 1. Introduction

The biological effects of vitamin D (VD) on target cells occur both by an extragenomic mechanism and by affecting the genome. The extragenomic activity of vitamin D is closely linked to the activation of tyrosine kinases and subsequent activation of the protein kinase C cascade. Genomic activity is mediated by the vitamin D receptor (VDR), which belongs to the nuclear steroid hormone receptor family. It acts as a ligand-dependent transcription factor that regulates the expression of sequences in the promoters of target genomes. Upon the attachment of vitamin D to the ligand-binding domain, VDR heterodimerizes with the retinoid X receptor (RXR), which is required for binding to a specific DNA sequence. VDR expression is observed not only in tissues, such as bones, the skin, intestines, and kidneys, but also in the brain, cardiac vascular smooth muscle, pancreas, parathyroid glands, adrenal glands, breasts, gonads, placenta, prostate, adipose tissue, and almost all tissues of the immune system. Numerous reports indicate the role of calcitriol in controlling the expression of over 200 genes [1,2,3].

VDR is encoded by a large gene localized on the long arm of chromosome 12 (locus 12q13.11), and it forms a heterodimer with RXRs. Both VDR and RXRs are members of the steroid nuclear superfamily. Polymorphisms of the *VDR* gene, or allelic variants, are common in the population, but their impact on the function of the VDR receptor has not yet been fully explained. Functional polymorphisms of the *VDR* gene include single-nucleotide polymorphisms (SNPs), such as those localized in the intron between exons 8 and 9: rs1544410 (Bsm-I), involving the change of guanine to adenine (G/A), rs7975232 (Apa-I), the change of thymine to guanine (T/G), detected by bsm1 and apa1 restriction enzymes, as well as rs731236 (Taq-I), with the change of thymine to cytosine (T/C), localized in exon 9 and detected by the taq1 enzyme. Another functional polymorphism is a cytosine-to-thymine substitution (C/T)―rs10735810 (Fok-I)―localized in exon 2, detected with the fok1 restriction enzyme. It is known to have a direct effect on the structure of the protein, as it causes a three-amino-acid shift in the translation start site, resulting in the synthesis of a receptor protein that is three amino acids shorter (424 aa), which is characterized by greater biological activity compared with the longer form (427aa) [4,5,6].

It is believed that these polymorphisms may be associated with changes in bone structure and increased risk of fractures [7,8,9]. VDR polymorphisms may also be associated with risk factors for cardiovascular disease, myocardial infarction [10,11,12], and type 2 diabetes [13,14,15,16,17]. A relationship has been observed between vitamin D deficiency and the occurrence of chronic diseases, including cardiovascular disorders. When calcitriol activates VDR, it affects the expression of genes closely related to the walls of blood vessels. They encode structural proteins, namely vascular endothelial growth factor and metalloproteinase, involved in vascular remodeling and the destabilization of atherosclerosis. They also affect the proteins taking part in the regulation of blood pressure. Through these mechanisms, vitamin D participates in most processes related to the pathogenesis of vascular diseases and cardiovascular disorders. The direct effect of vitamin D on the cardiovascular system results from its effect on blood vessels, the renin–angiotensin–aldosterone system, inflammatory processes, and the coagulation system [18].

Vitamin D may also have indirect effects on the cardiovascular system by affecting glucose and lipid metabolism and blood pressure [19,20,21].

Vitamin D levels, as well as VDR expression, decrease with age, which is of particular importance for postmenopausal women, who, due to the cessation of estrogen synthesis and the consequent lack of its protective effect, are at greater risk of reduced bone mass, cardiovascular disease, metabolic disorders, unfavorable lipid profiles, diabetes, and atherosclerosis with all of its long-term consequences [22,23,24]. In addition, serum antioxidant activity decreases during the aging process, thus increasing the risk of oxidative stress, which, in turn, is involved in the initiation of the inflammatory process [25,26].

Our work is a continuation of an earlier one in which we assessed the impact of selected adipokines, vitamin D deficiency, and sedentary lifestyle on the risk of metabolic disorders, leading to atherosclerosis and related to cardiovascular diseases in postmenopausal women [27].

The aim of the present study was to assess the relationships between VDR polymorphisms (*ApaI*, *BsmI*, *FokI*, and *TaqI*) and selected cytokines, inflammatory factors, and metabolic parameters in relation to the risk of metabolic disorders and atherogenicity in Polish postmenopausal women.

## 2. Materials and Methods

### 2.1. Study Design

This study was conducted on an ethnically homogeneous urban population of postmenopausal Caucasian Polish women from the city of Wroclaw, Lower Silesia Voivodeship, Poland. The women qualified for the study if they had their last menstruation (menopause) at least twelve months earlier. The study was intended to include nonsmokers of average health who were not currently receiving treatment and had not been treated in the past for serious diseases. Thus, the criteria for exclusion from the study were diabetes, cerebrovascular disease, liver or kidney disease, tobacco abuse, hormone replacement therapy, ovariectomy or hysterectomy, and the use of confounding drugs, such as those affecting carbohydrate or lipid metabolism. The final study sample comprised 321 postmenopausal women aged between 50 and 60 years. The study was approved by the Ethics Committee of Wroclaw University of Health and Sport Sciences (SKEBN 26/2016―September 2016). All participants gave written informed consent to their data being collected.

### 2.2. Anthropometrical and Biochemical Measurements

In accordance with the guidelines of the World Health Organization (WHO), all patients had their weight, height, and waist circumference (WC) measured. Their body mass index (BMI) and waist-to-hip ratio (WHR) were calculated according to the following formulas: BMI = body weight (kg)/height^2^ (m); WHR = WC (cm)/hip circumference (cm).

Between 8:00 a.m. and 10:00 a.m., after an overnight fast, venous blood samples of 10 mL were collected using standard blood-collection tubes for all biochemical and genetic tests. Serum and plasma samples were separated by centrifugation (3200 rpm for 15 min at room temperature). Serum was analyzed on the day of collection using commercial kits for glucose—colorimetric, total cholesterol (TC), high-density lipoprotein cholesterol (HDL-C), and triacylglycerol (TG) (Olympus Au 560, bioMerieux, Marcy l’Etoile, France). The levels of low-density lipid cholesterol (LDL-C) were calculated from the levels of TC, HDL-C, and TG using the Friedewald equation. The atherogenic index of plasma (AIP) was calculated as [log TG/HDL-C] and the Castelli Index as [(TC-HDL-C)/HDL-C]. Insulin concentrations were measured by a radioimmunoassay using the chemiluminescent method with commercial kits (IRMA of DPC). The homeostasis model assessment (HOMA) was calculated as [glucose (mmol/dL) × insulin (µIU/mL)/22.5]. The concentration of vitamin D as 25(OH)D was measured using RIA and intact PTH by IRMA with a test from Biosource Belgium, while sets from Millipore were used for the measurements of adiponectin and leptin. Additionally, we used commercial kits for TNF-α and IL-6 (IRMA, DIAsource ImmunoAssays, Ottignies-Louvain-la-Neuve, Belgium).

Total body fat (TF) was measured using dual-energy X-ray absorptiometry (DEXA). All scans were performed and analyzed using DPX + LUNAR and USA, and the Total Body software was used to determine the TF percentage [28].

Genomic DNA was isolated from peripheral blood leukocytes using the standard method (QIAmp DNA Mini Kit, Qiagen, Hilden, Germany). Genotyping in the area of the *VDR* gene polymorphisms *Bsm-I*, *Taq-I*, *Fok-I*, and *Apa-I* was carried out using polymerase chain reactions (PCR) and minisequencing (SNaPshot). The *VDR* gene fragments containing selected polymorphic sites were amplified using a TaKaRa Taq DNA polymerase amplification kit (Takara Bio, Shiga, Japan) in the presence of primers, as described by Lins et al. [29] (Table 1).

The PCR reaction mixture (20 µL) contained 10 µM primers, 1×PCR buffer containing 1.5 mM MgCl2, 200 µM dNTPs, 2 units of Taq polymerase, and 200 ng of genomic DNA. The reaction was carried out in a thermocycler (TPersonal Thermocycler, Biometra GmbH, Göttingen, Germany) under the following reaction conditions: initial denaturation at 95 °C for 5 min, followed by 35 cycles of denaturation at 95 °C for 30 s, primer annealing reaction at 58 °C for 30 s, primer extension at 72 °C for 60 s, and a final primer extension reaction at 72 °C for 10 min. The PCR reaction products were purified from an excess of primers and nucleotides (dNTPs) using a mixture of enzymes: alkaline phosphatase (SAP) and exonuclease I (ExoI) (Thermo Fisher Scientific, Waltham, MA, USA).

The minisequencing reaction (SNaPshot) was performed in a thermal cycler and consisted of 25 cycles including the following steps: denaturation at 96 °C for 10 s, primer annealing at 50 °C for 5 s, and DNA chain extension at 60 °C for 30 s. The minisequencing reaction was performed following the protocol of the ABI Prism SNaPshot Multiplex Kit (Thermo Fisher Scientific, Waltham, MA, USA) in the presence of primers described in the publication by Lins et al. [29], as shown in Table 2.

The reaction products were subjected to capillary electrophoresis in the ABI Prism 3100 Genetic Analyzer sequencer (Thermo Fisher Scientific) and analyzed using the GeneMapper Software v.4.0 computer software (Thermo Fisher Scientific).

The prevalence of *VDR* gene genotypes or alleles was examined (following the method of Hardy–Weinberg). We determined the between-genotype differences in body mass, fatty tissue content, glucose concentration, insulin concentration, lipid parameters, selected cytokine concentrations, insulin resistance index, insulin sensitivity index, and atherogenicity index.

### 2.3. Statistical Analysis

Statistical analysis was carried out using packages of the EPIINFO statistics program (v.3.5.2, dated 17 December 2010). For all groups, the following data were calculated: number of cases (N), mean values (X), and standard deviation (SD) of continuous parameters. In order to verify the hypothesis on the equality of particular trial mean values, an ANOVA analysis of variance was performed. For groups of nonhomogenous variance or with a small number of cases, the nonparametric Kruskal–Wallis test by ranks was used (the Bartlett test was used to check for homogeneity of variance); this is indicated by asterisks in Tables 4 and 5. For discrete parameters, the attribute prevalence was determined using the X^2^*df* test with Yates’ correction using an adequate number of degrees of freedom (*df*): *df* = (m − 1) × (*n* − 1), where m is the number of verses and *n* is the number of columns. In the case of statistically differences within three groups a post hoc type analysis was performed by the Bonferroni and Dunn’s post-hoc tests. A *p*-value below 0.05 was considered significant.

## 3. Results

The serum vitamin D concentrations for the group of 321 postmenopausal women were 24.1 ± 11.1 ng/mL; in 133 women, the concentration was below 20 ng/mL; in 110, it fell in the range from 20 to 29.9 ng/mL; and in 78 women, it was 30 ng/mL or higher. The serum level of PTH (parathormone) was 31.2 ± 14.4 pg/mL.

The mean age of menopause was 46.7 ± 2.8, the mean BMI was 27.3 ± 4.7 kg/m^2^, the mean waist circumference (WC) was 87.6 ± 11.6 cm, the mean hip circumference was 105.5 ± 9.4 cm, and the mean total fat (TF) was 37.5 ± 5.1%.

The frequency of *VDR* genotypes and the prevalence of the alleles *Bsm-I*, *Taq-I*, *Apa-I*, and *Fok-I* was consistent with the Hardy–Weinberg principle (Table 3).

The total number of samples for the *Bsm-I* genotype was 321. However, due to the degradation of DNA fragments containing the polymorphic sites analyzed, it was not possible to determine all polymorphisms for the *Taq-I*, *Apa-I*, and *Fok-I* genotypes, for which the number of samples was 291.

Table 4 shows the anthropometric and metabolic risk factors for chronic metabolic inflammation on each *Bsm-I* genotype of the *VDR* gene in the study group.

Postmenopausal females with the GG *Bsm-I* genotype seemed to have higher HOMA values than those with the GA and AA genotypes. Scores of multiple comparison corrections between genotypes were significant for HOMA values (GA vs. GG); total cholesterol (GA vs. AA); TG concentration and AIP (GA vs. AA).

Moreover, carriers of the GG genotype tended to have lower adiponectin concentrations than those with GA, and especially those with AA, but there was no statistical difference (*p* = 0.08). However adiponectin concentration value between GG vs. AA was statistically significant.

AA *Bsm-I* genotype carriers showed significantly higher values of LDL-C, LDL-C/HDL-C, and Castelli indices than the GG and GA genotypes carriers, respectively (*p* < 0.014, 0.009, and 0.008). This may suggest that carriers of the GG genotype may be prone to insulin resistance, and carriers of the AA genotype have a more atherogenic lipid profile of blood serum. In summary, this may indicate a relationship between *Bsm-I* polymorphism and the development of arteriosclerosis or cardiovascular disease.

Table 5 shows the values of metabolic risk factors for the individual *Taq-I* genotypes of *VDR* in the study group.

Postmenopausal women with the TT genotype had statistically significantly higher HOMA insulin resistance indices (*p* < 0.041) and significantly lower adiponectin serum concentrations than women with the TC and CC genotypes (*p* < 0.032). Significant scores for multiple comparison we found only for HOMA (TT vs. TC) and adiponectin concentration value (TT vs. CC; TC vs. CC). This would indicate that the TT Taq-I genotype is more strongly related to insulin resistance than the TC and CC genotypes, and is, therefore, more metabolically unfavorable.

The anthropometric risk factors did not significantly differ by the genotype of the *Taq-I* polymorphism, and *Bsm-I* similarly.

The *Fok-I* and *Apa-I* polymorphisms did not show any relation to anthropometric or metabolic risk factors for low-grade chronic metabolic inflammation.

## 4. Discussion

### 4.1. The Prevalence of VDR Polymorphism

The frequency of individual vitamin D receptor polymorphisms was similar to that observed by other authors for *Bsm-I* and *Fok-I* in both the Lower Silesian [11] and general Polish populations [30,31,32], as well as for other Caucasian populations [10,33]. The occurrence of genotypes and the allele frequency of the *Taq-I* polymorphism in our menopausal women were very similar to those observed in the Austrian population [34], in contrast to other populations, such as those from the Middle East (Syria, Jordan, and Iran) [34] and the United States (Minnesota whites, African Americans in Pennsylvania, and Mexicans in California) [35]. The data indicate that the racial and ethnic factor has great significance in the distribution of genotypes and the frequency of alleles of the *VDR* receptor gene in healthy populations.

### 4.2. VDR Polymorphism and Anthropometric Risk Factors of Metabolic Diseases

We found no significant differences between anthropometric risk factors, such as BMI, WC, WHR, and TF%, across the *Bsm-I*, *Apa-I*, *Taq-I*, and *Fok-I* genotypes in our postmenopausal group. Similarly, Łaczmański et al. [30] did not observe a relationship between the *Fok-I* and *Bsm-I* polymorphisms and BMI, waist circumference, and WHR in a group of Polish 881 women and men over 65 years of age. Kaleta et al. [36], who studied 151 men and women with morbid obesity, also did not find a relationship between *Bsm-I* and *Fok-I* polymorphisms and BMI. Other authors reported similar findings for *Fok-I* [11,37]. Similar to our results, Ochs-Balcom et al. [38] did not find any association of *Bsm-I* and *Fok-I* polymorphisms with BMI, waist circumference, or abdominal fat content in a population of white American women. On the other hand, Grundberg et al. [39] noticed a link between the AA *Bsm-I* genotype and higher body weight and adipose tissue content in premenopausal women in a Swedish population. Zhao et al. came to conclusions opposite to ours [40], finding a significant relationship between *Fok-I* and BMI and *Bsm-I* and WC in a northern Chinese population (*n* = 1169). The relationship of the individual *Fok-I* and *Bsm-I*, *Apa-I*, and *Taq-I* polymorphisms with anthropometric risk factors of metabolic disorders remains unclear, and the results of the studies in the literature are inconclusive.

### 4.3. VDR Gene Polymorphism and Metabolic Risk Factors 

The results of our research indicate that postmenopausal women carriers of the *Taq-I* polymorphism TT genotype show statistically significantly higher HOMA values (*p* < 0.041) and significantly lower adiponectin concentrations (*p* < 0.032) than carriers of the other genotypes. This is an interesting observation that we have not seen reported in the literature available to us. Vitamin D is known to be involved in the regulation of insulin secretion and may affect insulin sensitivity [37,41,42]. The results of some studies suggest a relationship between VDR polymorphisms and insulin resistance, but this particularly associates the *Bsm-I* polymorphism with type 2 diabetes [43,44].

Our results do not relate the *Fok-I* and *Bsm-I* polymorphisms to insulin resistance. Similarly, Kaleta et al. [36] did not note an association of *Bsm-I* and *Fok-I* polymorphisms with hyperglycemia and dyslipidemia in a group of morbidly obese Polish patients, but a separate study in an elderly Polish population showed a significantly higher insulin concentration and HOMA in carriers of the AA genotype of the *Bsm-I* polymorphism and pointed to a relationship between the *Fok-I* polymorphism and glucose, insulin concentrations, and lipid parameters [30]. Małecki et al. [32] did not confirm the relationship between glycemia and any of the four VDR polymorphisms we studied (*Bsm-I*, *Taq-I*, *Apa-I*, and *Fok-I*). Schuch et al. [19] did not report any relationship between VDR polymorphism and glycemia, HOMA, or triglycerides in a Brazilian population. Contrary to the above observations, our results indicate that the *Taq-I* polymorphism is related to insulin resistance, with the TT genotype presenting the highest value of HOMA in postmenopausal women.

Few studies have assessed the relationship between *VDR* polymorphisms and the lipid profile. Our results indicate that there is a relationship between the *Bsm-I* polymorphism and the concentration of LDL-chol and atherogenicity indices in healthy postmenopausal women. Postmenopausal women with the AA *Bsm-I* genotype had statistically significantly higher concentrations of the LDL-chol fraction (*p* < 0.0135), indices of atherogenicity (LDL-chol/HDL-chol (*p* < 0.009), and the Castelli index (*p* < 0.008) than those with the GA and GG genotypes, which may indicate that this genotype is associated with a more atherogenic serum lipid profile and, therefore, with a higher risk of developing metabolic disorders and atherosclerosis. Similarly, higher levels of LDL-chol in carriers of the AA genotype were observed by Phabphal and Geater [45], who investigated the relationship of *Bsm-I* polymorphism with vascular risk and metabolic syndrome in epilepsy patients treated with valproate. The authors did not find any relationship between that polymorphism and the concentrations of insulin, glucose, or HOMA. Karonova et al. [46] found a relationship between the *Bsm-I* and *Apa-I* variants and an atherogenic lipid profile in middle-aged Russian women. GG carriers of *Bsm-I* showed significantly higher TG levels, whereas TT and TG *Apa-I* carriers had significantly higher TG and LDL-C. Contrary to our observations, Filus et al. [11] reported a relationship between the *Fok-I* polymorphism and the concentration of HDL-C in male carriers of the FF genotype, who presented significantly lower concentrations of HDL-chol than those with the ff genotype. Similar observations were presented by Schuch et al. [19] for a Brazilian population and Zhao et al. [40] for a northern Chinese population; they showed a relationship between the *Fok-I* polymorphism and the concentration of HDL-chol, especially in the case of ff genotype carriers, who had significantly lower concentrations of this lipid fraction. The results of our study did not support the relationship between *Fok-I* and *Apa-I* and the HDL-chol concentration, or with other lipid parameters or indicators of atherogenicity in postmenopausal women. As in the relationship between *VDR* genotypes and anthropometric risk factors, the evidence for metabolic factors increasing metabolic disturbances remains inconclusive [17,46,47,48].

In conclusion, we found associations between *Bsm-I* and *Taq-I* polymorphisms and metabolic risk factors in postmenopausal women. Postmenopausal women with the AA genotype of *Bsm-I* showed significantly higher concentrations of the LDL-C fraction and higher atherogenicity indices (LDL-chol/HDL-chol and the Castelli index) than women with the GA and GG genotypes. This may indicate that the AA genotype of the *Bsm-I* polymorphism is associated with a more atherogenic serum lipid profile and that its carriers are, therefore, more likely to develop atherosclerosis and cardiovascular disease than those of the other two genotypes. Similarly, postmenopausal women with the TT *Taq-I* genotype had significantly higher insulin resistance, as measured by HOMA (*p* < 0.0412), than women with the TC and CC genotypes. They also had significantly lower serum levels of adiponectin (*p* < 0.032) than women with the other two genotypes. This allows us to conclude that the TT genotype of this polymorphism is metabolically less favorable and may be associated with insulin resistance, and is, therefore, more atherogenic. No association was found between the *Bsm-I* and *Taq-I* polymorphisms and anthropometric risk factors for metabolic disturbances in postmenopausal women. Furthermore, the *Fok-I* and *Apa-I VDR* polymorphisms were not related to anthropometric and metabolic risk factors for cardiovascular disease.

Strength and limitations: Further research on a larger group of women is needed. One strength of our study is that we assessed a very homogeneous group of women aged 50–60. Furthermore, the frequency of the *VDR* receptor gene polymorphisms was similar to that observed by other researchers in the Lower Silesian and Polish populations, and was also comparable to the frequency of these polymorphisms found in other Caucasian populations. Thus, our observations allowed us to determine the genotype of *VDR* polymorphisms associated with increased risk of atherosclerosis and insulin resistance. The limitations of our study are the lack of haplotype analysis. The haplotype analysis wasn’t performed, due several technical problems. Furthermore only a few post-hoc analyses were found with the power desired 80% and they are indicated in the Results. 

## 5. Conclusions

*Taq-I* and *Bsm-I* vitamin D receptor gene polymorphisms are associated with the atherogenic serum lipid profile and metabolic disorders, which may increase the risk of atherosclerosis and cardiovascular disease in an ethnically homogenous postmenopausal population of women from Poland.

## Figures and Tables

**Table 1:** Sequence of primers for PCR.

Polymorphism	Forward Primers for PCR (5′-3′)	Reverse Primers for PCR (3′-5′)	Size of the PCR Product (bp)
FokI	GGCCTGCTTGCTGTTCTTAC	TCACCTGAAGAAGCCTTTGC	174
BsmI	CCTCACTGCCCTTAGCTCTG	CCATCTCTCAGGCTCCAAAG	209
ApaI	CTGCCGTTGAGTGTCTGTGT	TCGGCTAGCTTCTGGATCAT	242
TaqI			

**Table 2:** Sequence of primers for minisequencing reactions.

Polymorphism	Forward Primers for PCR (5′-3′)
FokI	(T)_31_GCTGGCCGCCATTGCCTCC
BsmI	(T)_21_CAGAGCCTGAGTATTGGGAATG
ApaI	(T)_12_GTGGTGGGATTGAGCAGTGAGG
TaqI	(T)_9_GCGGTCCTGGATGGCCTC

**Table 3:** VDR gene polymorphisms in the study group.

Genotype Polymorphism	Genotype	*n* (Observed)	*n* (Expected)	p	q	*χ* ^2^	*p*
N = 321							
*Bsm-I* (rs1544410) G < A	GG GA AA	**121** (37.71%) 163 (50.8%) 37 (11.5%)	127.1 (39.8%) 149.5 (46.6%) 43.7 (13.6%)	G 0.63	A 0.37	2.6136	0.106
*Taq-I* (rs731236) T < C	TT TC CC	122 (41.9%) 133 (45.7%) 36 (12.4%)	122.1 (42.0%) 132.8 (45.6%) 36.1 (12.4%)	T 0.65	C 0.35	0.0007	0.979
*Apa-I* (rs7975232) T < G	TT TG GG	59 (20.4%) 153 (52.5%) 79 (27.1%)	63.1 (21.7%) 144.8 (4.8%) 83. (28.5%)	T 0.47	G 0.53	0.9302	0.334
*Fok-I* (rs10735810) C < T	CC CT TT	99 (34%) 148 (50.9) 44 (15.1%)	102.8 (35.4%) 140.3 (48.2%) 47.8 (16.4%)	C 0.41	T 0.59	0.8759	0.349

G: guanine; A: adenine; T: thymine; C: cysteine; p: frequency of alleles encoding G, T, T, and C; q: frequency of alleles encoding A, C, G, and T.

**Table 4:** Anthropometric and metabolic risk factors in the study group by the *Bsm-I* genotype of the *VDR* gene.

Risk Factor of Metabolic Disturbances	Genotype GA *n* = 163	Genotype GG *n* = 121	Genotype AA *n* = 37	*p*
BMI (kg/m^2^)	27.2 ± 4.9	27.4 ± 4.9	27.5 ± 4.7	0.944
WC (cm)	89.1 ± 11.5	87.7 ± 11.7	89.4 ± 11.5	0.570
Hip circumference (cm)	105.1 ± 10	105.8 ± 8.7	105.9 ± 8.8	0.536
WHR (cm/cm)	0.830 ± 0.078	0.828 ± 0.075	0.835 ± 0.063	0.877
TF (%)	37.5 ± 54	37.6 ± 5.0	37.3 ± 0.0	0.956
Glucose (mg/dL)	87.7 ± 9.6	88.8 ± 9.9	89.7 ± 8.2	0.49
Insulin (µIU/mL)	6.34 ± 4.30	6.51 ± 4.09	6.94 ± 3.64	0.73
HOMA	1.30 ± 0.81	1.57 ± 1.11	1.44 ± 0.95	0.06
T-chol (mg/dL)	240.0 ± 38.6	248.1 ± 45.7	257.1 ± 43.9	0.054
HDL-C (mg/dL)	72.1 ± 17.7	69.5 ± 17.0	66.7 ± 16.1	0.170
LDL-C (mg/dL)	148 ± 36.1	156.4 ± 41.2	167.6 ± 40.2	0.014 *
TG (mg/dL)	100.8 ± 43.5	112.2 ± 50.1	114.3 ± 45.2	0.072
LDL-C/HDL-C	2.20 ± 0.82	2.40 ± 0.89	2.65 ± 0.92	0.009 *
Castelli index	2.50 ± 0.95	2.76 ± 1.05	3.02 ± 1.05	0.008 *
AIP	0.280 ± 0.556	0.409 ± 0.612	0.494 ± 0.545	0.055
TNF-α (pg/mL)	15.2 ± 19.1	19.7 ± 25.4	16.5 ± 19.1	0.22
IL-6 (pg/mL)	22.4 ± 64.3	15.5 ± 7.6	17.6 ± 7.2	0.44
Adiponectin (µg/mL)	14.3 ± 6.0	13.5 ± 6.3	16.6 ± 8.2	0.08
Leptin (ng/mL)	12.0 ± 6.7	12.5 ± 7.1	13.0 ± 7.6	0.70

BMI: body mass index; WC: waist circumference; WHR: waist-to-hip ratio; TF: total fat; HOMA: homeostasis model assessment; T-chol: total cholesterol; HDL-C: HDL-cholesterol; LDL-C: LDL-cholesterol; Castelli index: (T-chol-HDL-C)/HDL-C; AIP atherogenic index of plasma; TNF-α: tumor necrosis factor; IL-6: interleukin-6; * statistically significant (*p* < 0.05).

**Table 5:** Anthropometric and metabolic risk factors in the study group by the *Taq-I* genotype of the *VDR* gene.

Parameter Risk Factor	Genotype TT *n* = 133	Genotype TC *n* = 122	Genotype CC *n* = 36	*p*
BMI (kg/m^2^)	27.8 ± 4.8	27.4 ± 4.8	26.7 ± 4.0	0.48
WC (cm)	88.4 ± 11.6	88.2 ± 11.6	86.5 ± 10.9	0.67
Hip circumference (cm)	106.1 ± 9.1	105.5 ± 10.4	105.1 ± 7.5	0.79
WHR	0.832 ± 0.069	0.837 ± 0.082	0.822 ± 0.068	0.657
TF (%)	37.8 ± 5.1	38.0 ± 4.9	36.5 ± 5.1	0.36
Glucose (mg/dL)	90.0 ± 11.9	87.6 ± 9.6	87.1 ± 7.5	0.132
Insulin (μIU/mL)	6.86 ± 4.68	6.35 ± 4.02	5.99 ± 3.48	0.451
HOMA	1.55 ± 1.10	1.26 ± 0.75	1.31 ± 0.98	0.041 *
T-chol (mg/dL)	249.2 ± 47.1	239.9 ± 36.6	248.3 ± 39.9	0.199
HDL-C (mg/dL)	70 ± 17.4	70.5 ± 18.1	70.5 ± 14.2	0.968
LDL-C (mg/dL)	156.6 ± 42.8	149.2 ± 34.2	157.3 ± 39.8	0.267
TG (mg/dL)	113.0 ± 53.0	103.3 ± 40.3	102.8 ± 43.1	0.518
LDL-C/HDL-C	2.39 ± 0.92	2.27 ± 0.82	2.35 ± 0.89	0.567
Castelli index	2.75 ± 1.07	2.60 ± 0.96	2.67 ± 1.02	0.482
AIP	0.403 ± 0.625	0.340 ± 0.550	0.315 ± 0.539	0.598
TNF-α (pg/mL)	17.1 ± 24.0	17.1 ± 21.2	15.6 ± 16.0	0.881
IL-6 (pg/mL)	20.7 ± 63.9	18.5 ± 33.6	16.6 ± 4.0	0.881
Adiponectin (µg/mL)	13.8 ± 6.2	13.8 ± 5.9	16.7 ± 6.7	0.032 *
Leptin (ng/mL)	12.9 ± 7.3	12.0 ± 6.6	12.3 ± 7.4	0.625

BMI: body mass index; WC: waist circumference; WHR: waist-to-hip ratio; TF: total fat; T-chol: total cholesterol; HOMA: homeostasis model assessment; Castelli index: (T-chol-HDL-C)/HDL-C; AIP atherogenic index of plasma; TNF-α: tumor necrosis factor; IL-6: interleukin-6; * statistically significant (*p* < 0.05).
